# Tailoring Cobalt Content in MnO_*x*_ Nanowires for Superior Supercapattery Performance

**DOI:** 10.1021/acsomega.5c12806

**Published:** 2026-02-27

**Authors:** Fernando José Soares Barros, Samuel da Silva Eduardo, Klebson Lucas Pereira Cardozo, Hector A. Aguilar Vitorino, Carlos Martins Aiube, Mariana Lumi Ichihara Sado, Camila de Lima Ribeiro, Paulo Eduardo Narcizo de Souza, Alysson Martins Almeida Silva, Auro Atsushi Tanaka

**Affiliations:** † Department of Chemistry, 37892 Federal University of Maranhão, Av. dos Portugueses, 1966, São Luís, MA 65080-805, Brazil; ‡ Department of chemistry, 28125 Universidade Federal do Rio de Janeiro, Avenida Athos da Silveira Ramos, n° 149, 21941-909, Rio de Janeiro, RJ 21941-901, Brazil; § Department of Fundamental Chemistry, Institute of Chemistry, University of São Paulo, Av. Prof. Lineu Prestes, 748, São Paulo, SP 05508-000, Brazil; ∥ Institute of Chemistry, 28127 University of Brasília, Campus Universitário Darcy Ribeiro, Asa Norte, Brasília, DF 70910-900, Brazil; ⊥ Department of Mechanical Engineering, University of Brasilia, Campus Universitário Darcy Ribeiro, Asa Norte, Brasília, DF 70910-900, Brazil; # Institute of Physics, University of Brasilia, Campus Universitário Darcy Ribeiro, Asa Norte, Brasília, DF 70910-900, Brazil

## Abstract

Herein, we report the urea-assisted synthesis of Co-doped MnO_*x*_ nanowires with cobalt contents of 5.85, 6.63, and 19.22 wt %. ICP-OES confirmed Co incorporation, while SEM, TEM, and EDS analyses showed that low-to-moderate Co loadings preserve the nanowire morphology with homogeneously dispersed Co species, whereas high Co content induces aggregation. XPS revealed that 6.63 wt % Co optimized the Mn^4+^/Mn^3+^ ratio and increased the concentration of defect-related surface oxygen species, while excessive Co doping promoted Mn reduction. EPR/FMR measurements confirmed the formation of metallic Co aggregates at higher loadings. Electrochemical testing in 2 M KOH demonstrated that Co-MnO_*x*_(6.63 wt %) delivered the highest specific capacitance (1468.65 F·g^–1^ at 1 A·g^–1^) and excellent cycling stability. Moreover, a AC//Co-MnO_*x*_(6.63 wt %) supercapattery achieved an energy density of 393.6 Wh·kg^–1^ and a power density of 2928 W·kg^–1^ with 61% retention after 2200 cycles. The enhanced electrochemical activity is attributed to an optimized cobalt content that maximizes defect-related oxygen species and promotes favorable Co–Mn electronic interactions without inducing surface saturation, resulting in improved supercapattery performance.

## Graphical abstract

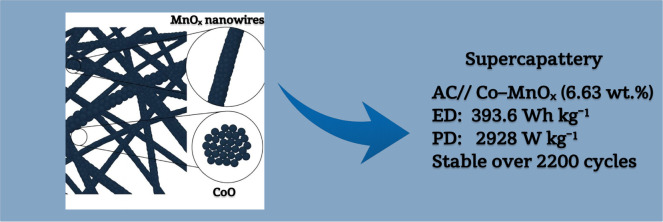

## 1 Introduction

The intermittency of renewable energy sources makes efficient energy storage systems essential for reliable power delivery.^1^ Electrochemical energy storage devices have therefore attracted research interest as solutions for balancing energy generation and consumption,^2^ with supercapacitors (SCs) standing out due to their high power density.^1,3,4^ Within this context, supercapatteries, also classified as hybrid supercapacitors (HSCs), have been developed to bridge the performance gap between conventional SCs and rechargeable batteries.^5–7^ Unlike electric double-layer capacitors EDLCs and pseudocapacitors, which rely on a single charge-storage mechanism, supercapatteries combine both by pairing a battery-type positrode with an EDLC-type negatrode, delivering high power density, moderate-to-high energy density, and cycling stability.^6,7^

The electrochemical performance of these devices depends on the properties of the electrode materials and can be enhanced through strategies such as nanostructuring, compositing, and surface functionalization.^7^ Promising candidates for supercapacitor electrodes include metal oxides, metal hydroxides, metal disulfides, and layered hydroxides.^5,6,8^ Among them, manganese oxides (MnO_*x*_), have attracted attention in catalysis, energy storage, and electrochemical sensors, owing to their low cost and natural abundance.^9–11^ Their ability to undergo valence transitions among Mn^2+^, Mn^3+^, and Mn^4+^ facilitates electron transfer efficiency, making them suitable for electrochemical energy storage systems.^10^ For supercapacitors, MnO_*x*_ represents an alternative to noble metal oxides due to its theoretical capacitance (up to 1370 F·g^–1^); however its practical application is often limited by poor ionic conductivity and low surface area.^12^ To overcome these challenges, the incorporation of transition-metal dopants, such as cobalt, has been widely explored.

The combination of cobalt and manganese oxides in bimetallic structures enhances electrochemical performance by uniting the high oxidation potential of cobalt with the superior electron mobility of manganese.^13^ These advantages have stimulated interest in Co-doped MnO_*x*_ for supercapacitor electrodes, prompting diverse synthesis approaches to optimize charge-storage behavior. For instance, Co-doped MnO_*x*_ nanorods produced via incipient wet impregnation exhibited a specific capacitance of 1478.52 F·g^–1^ at 1 A·g^–1^,^5^ while hierarchical Co-doped MnO_*x*_ nanowires synthesized hydrothermally, delivering strong performance in hybrid supercapacitors.^14^ Microwave-assisted cobalt–manganese oxides have also been reported, achieving 323 F·g^–1^ at 0.5 A·g^–1^.^13^ Beyond oxides, cobalt manganese phosphate thin films prepared hydrothermally reached 571 F·g^–1^ at 2.2 A·g^–1^.^15^

Several techniques have been reported for incorporating cobalt into diverse host matrices. In particular, cobalt modification of MgAl layered double hydroxides by impregnation with cobalt­(II) acetylacetonate has been shown to effectively tune structural and surface properties.^16^ Urea-assisted synthesis has also been widely employed to produce high-surface area cobalt–aluminum layered double hydroxides for energy storage applications,^17^ as well as to generate cobalt nanoparticles encapsulated in nitrogen-doped carbon nanotubes via urea-assisted pyrolysis, yielding conductive hierarchical structures with promising bifunctional activity for the hydrogen evolution reaction.^18^

In this work, we report the development of a Co-doped MnO_*x*_ electrode material via a urea-assisted doping approach, followed by comprehensive physicochemical characterization. Emphasis is placed on spectroscopic techniques, including XPS and EPR, to investigate elemental composition, oxidation states, and defect structures. The electrode material was evaluated in both a symmetric supercapacitor and a supercapattery configuration, using activated carbon as the negative electrode.

## 2 Experimental Section

### 2.1 Synthesis of MnO_*x*_ Nanowires

In a typical procedure, 0.4 g of MnSO_4._H_2_O (99%, Sigma-Aldrich) and 1.0 g of KMnO_4_ (99%, Sigma-Aldrich) were dissolved in 30 mL of deionized water and transferred into a 100 mL Teflon-lined stainless steel autoclave. The autoclave was heated to 140 °C and maintained under stirring for 19 h. After naturally cooling to room temperature, the resulting nanowires were washed three times with ethanol (15 mL, 95%, Vetec) and three times with water (15 mL) using successive centrifugation and decantation steps. Finally, the product was dried at 80 °C for 6 h in air.

### 2.2 Cobalt Deposition onto MnO_*x*_ Nanowires by Urea Hydrolysis

The method proposed by Eschemann et al. was adapted for the incorporation of cobalt into the MnO_*x*_ nanowires.^19^ Urea hydrolysis was used to prepare Co/MnO_*x*_ electrode materials with 5–20 wt % cobalt loadings as follows: 10 mg of MnO_*x*_ nanowires were dispersed in 75 mL of an aqueous solution containing the appropriate amount of CoCl_2_·6H_2_O (98%, Sigma-Aldrich). The pH was adjusted to 3 using 0.1 M HNO_3_ (65–70% Synth) and the suspension was heated to 90 °C under vigorous stirring. Subsequently, 5 mL of an aqueous urea solution (0.45 g in 5 mL, Dinâmica, Brazil) was added. The suspension was stirred for 4 h and then allowed to cool to room temperature. The resulting solid was filtered and dried at 60 °C overnight. Thermal treatment was carried out under a nitrogen flow at 400 °C for 4 h (heating rate: 25 °C/min). The resulting samples were labeled as Co-MnO*_x_* (*A* wt %), where *A* is the Co content.

### 2.3 Measurements and Characterization

Elemental analysis was carried out by Inductively Coupled Plasma Optical Emission Spectrometry (ICP-OES) (Spectro Arcos, SPECTRO Analytical Instruments GmbH, Germany), with a detection limit of 0.01 ppm. Samples were digested using aqua regia (HNO_3_:HCl, 1:3) and heated at 100 °C in sealed tubes using a digestion block.

The surface morphology of the materials was examined with a JEOL JSM-7100F Field Emission SEM, equipped with Electron Dispersive X-ray Spectroscopy system (EDS) (EX-37270VUP) for elemental mapping. The size distribution of cobalt particles was determined by measuring 200 particles from each sample micrograph using ImageJ software. The resulting data were then used to construct histograms, and the particle size distributions were fitted to a LogNormal function with the software Origin 2018 to calculate the average grain size values.

Transmission electron microscopy (TEM) imaging was performed with a JEOL JEM-2100. For TEM sample preparation, the materials were dispersed in isopropanol under ultrasonication, and a drop of the suspension was deposited onto carbon-coated copper grids with a lacey carbon film. Nanowire dimensions were determined from these images using ImageJ software. For each sample, 20 individual nanowires were analyzed to obtain the average length and thickness. Representative TEM images illustrating the measurement procedure are provided in the Supporting Information (Figure S1).

X-ray photoelectron spectroscopy (XPS) analyses were carried out using a Thermo Fisher K-Alpha instrument (East Grinstead, UK) equipped with a monochromatic Al Kα source (photon energy = 1486.6 eV). High-resolution spectra were collected at a pass energy of 0.05 eV, and 20 to 30 scans were acquired per element to improve the signal-to-noise ratio.

Magnetic resonance measurements were carried out on a Bruker EMXplus spectrometer (X-band, 9 GHz), equipped with a high-sensitivity ER 4119HS cavity. The experimental parameters were: microwave power of 2.0 mW, modulation amplitude/frequency of 10 G/100 kHz, and magnetic field sweep from 0 to 9500 G at room temperature.

### 2.4 Electrochemical Characterization

Electrochemical analyses were performed using an Autolab PGSTAT302N potentiostat/galvanostat (Metrohm, The Netherlands), and the data were evaluated using NOVA 2.1.4 software. A conventional three-electrode setup was employed, consisting of an Ag/AgCl reference electrode, a platinum wire as the counter electrode, and a nickel foam working electrode (area: 1.5 cm^2^) coated with the active material.

To prepare the working electrode, the slurry combined the active material, Super P carbon black (Super P, >99%, Alfa Aesar), and a PVDF (average *M*_w_ ∼ 534,000, Sigma-Aldrich) solution in *N*-methyl-2-pyrrolidone (NMP, 99.5%, Sigma-Aldrich) in a mass ratio of 8:1:1. The mixture was homogenized using an agate mortar for 10 min. A volume of 20 μL of this ink was drop-cast onto the nickel foam and dried at 60 °C for 4 h. The electrode was then affixed to a nickel wire for use in electrochemical measurements. All tests were conducted in a 2 M KOH (99%, Isofar) aqueous electrolyte at room temperature.

Cyclic voltammetry (CV) was carried out across a potential window from −0.2 to 0.5 V, using scan rates of 5, 10, 20, 40, and 80 mV·s^–1^. Galvanostatic charge–discharge (GCD) experiments were conducted at current densities between 1 and 20 A·g^–1^. The cycling durability of the electrode was evaluated through 1,000 consecutive GCD cycles. In addition, electrochemical impedance spectroscopy (EIS) was conducted before and after cycling, within the frequency range of 0.1 to 1000 Hz.

A two-electrode configuration was also used to assemble a device, where one electrode contained the active material and the other contained activated carbon (AC). Both electrodes were fabricated using the same method as described above. To ensure the mass balance, the required amount of AC was estimated based on specific capacitance values from prior electrochemical tests conducted on carbon-only electrodes. The mass ratio between electrodes was determined using the charge balance expression ([Disp-formula eq1]):

1 $$\frac{m^{+}}{m^{-}}=\frac{Q^{+}}{Q^{-}}=\frac{m\times C_{s}\times \Delta V}{m\times C_{s}\times \Delta V}$$

## 3 Results and Discussion

### 3.1 Characterization of Electrode Materials

ICP-OES confirmed that the cobalt contents in the synthesized solids were 5.85, 6.63, and 19.22 wt % for nominal loadings of 5, 10, and 20 wt %, respectively. Thus, the materials were labeled as Co-MnO_*x*_(5.85 wt %), Co-MnO_*x*_(6.63 wt %) and Co-MnO_*x*_(19.22 wt %). These results indicate a slightly higher incorporation than expected for the 5 wt % sample and a lower incorporation for the 10 wt % sample, suggesting a nonlinear relationship between the precursor concentration and final metal content. In contrast with our previous work, Co-doped MnO_*x*_ nanorods using incipient wet impregnation, reporting significantly lower incorporat**i**on efficiencies under the same nominal conditions: 1.37, 4.79, and 10.19 wt % Co for 5, 10, and 20 wt % nominal additions, respectively.^5^ The superior metal uptake observed in this study suggests that urea hydrolysis offers more favorable conditions for cobalt incorporation, due to the gradual increase in pH that promotes homogeneous precipitation and better interaction with the high surface area of MnO_*x*_ nanowires. Moreover, differences in morphology between nanowires and nanorods may also influence metal anchoring, as nanowires tend to exhibit a more accessible surface and higher defect density, which facilitates cobalt binding.

The TEM images of Co-doped MnO_*x*_ nanowires highlight their morphological evolution as a function of cobalt loading (Figure 1). At lower cobalt content (5.85 wt %, Figure 1A–C), the MnO_*x*_ nanowires exhibit well-defined, elongated structures with an average length of 465.48 nm and a thickness of 10.24 nm, together with discrete, homogeneously distributed cobalt oxide particles. Increasing the Co loading to 6.63 wt % (Figure 1D–F) results in a greater density of Co-containing particles on the nanowire surfaces, accompanied by some degree of nanoparticle aggregation, concurrently, the nanowires show an average length of 487.13 nm and an increased thickness of 231.09 nm, indicating substantial surface coverage by cobalt species while the overall wire-like morphology is preserved. At the highest cobalt content (19.22 wt %, Figure 1G–I), significant agglomeration of cobalt oxide particles is observed, partially masking the nanowire surfaces. These nanowires display an increased average length of 656.71 nm and a thickness of 154.78 nm, suggesting saturation of anchoring sites and extensive particle growth or aggregation. While intimate contact between the Co particles and MnO_*x*_ is still evident, the increased surface coverage at higher loadings may reduce the accessibility of active MnO_*x*_ sites, potentially impacting electrochemical performance. These morphological differences suggest that cobalt distribution and particle dispersion are strongly dependent on the nominal loading and influence the structural integrity of the hybrid material.

**Figure 1:**
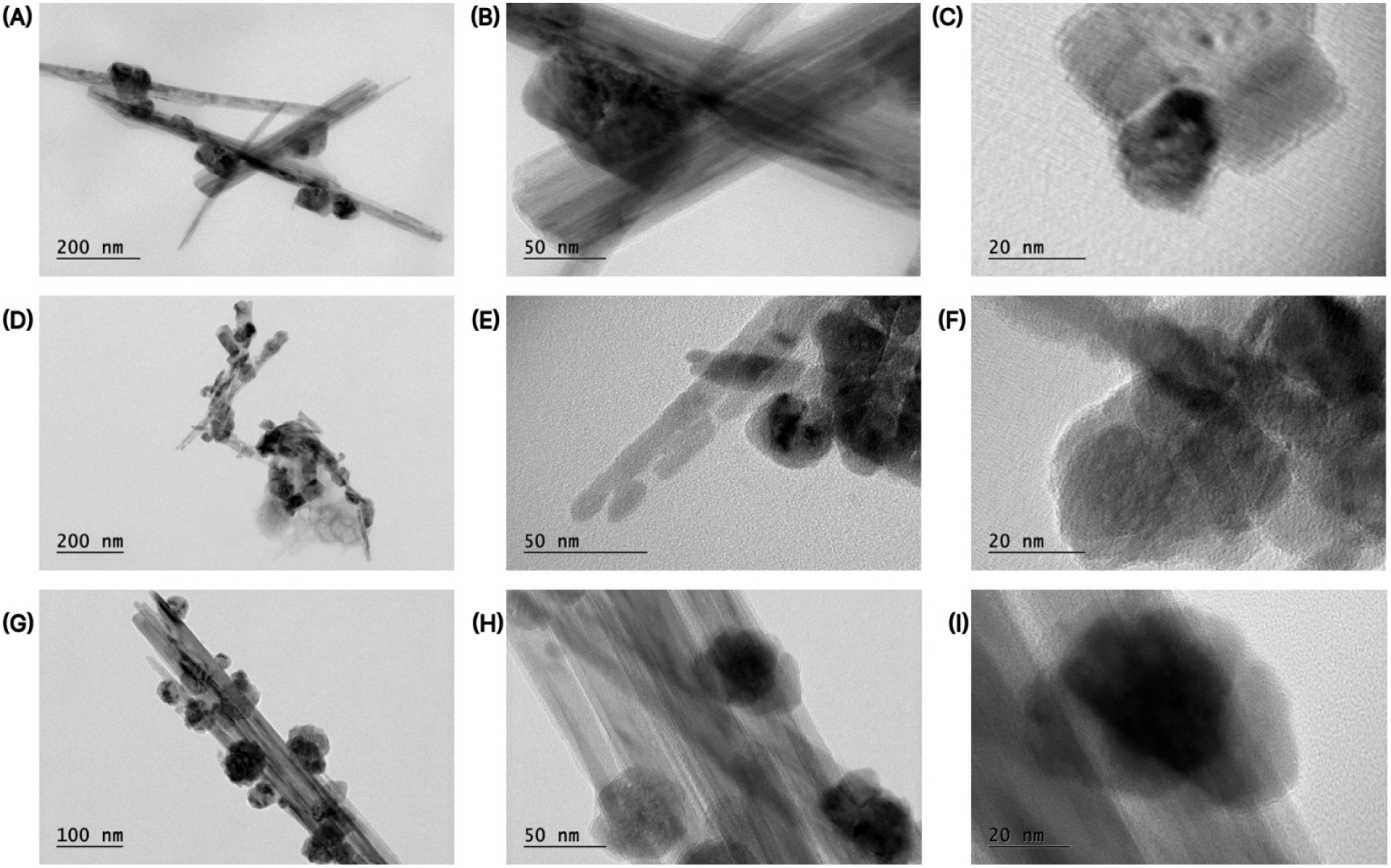
TEM images of Co-impregnated MnO*_x_* nanowires synthesized via urea hydrolysis. (A–C): Co-MnO*_x_* (5.85 wt %); (D–F): Co-MnO*_x_* (6.63 wt %); (G–I): Co-MnO*_x_* (19.22 wt %).

The SEM and EDS results in Figure 2 are consistent with the ICP-OES measurements and TEM images discussed earlier (Figure 1), confirming the influence of cobalt content on the morphology and elemental distribution of the Co/MnO_*x*_ nanowire composites. For 5.85 wt % Co (Figure 2A–D), the SEM images reveal a preserved nanowire-like structure with minor surface roughening and an average size of the Co particles of approximately 0.21 μm. The high-resolution SEM (Figure 2C) and EDS map (Figure 2D) show a clear localization of cobalt along the MnO_*x*_ nanowires, with no evidence of large aggregates. This corroborates the TEM observations (Figure 1A–C), where cobalt oxide nanoparticles are discretely anchored to the wire surfaces. The ICP-OES result confirms a near-nominal incorporation, indicating that urea hydrolysis at this loading facilitates effective and homogeneous deposition of cobalt without disrupting the MnO_*x*_ morphology.

**Figure 2:**
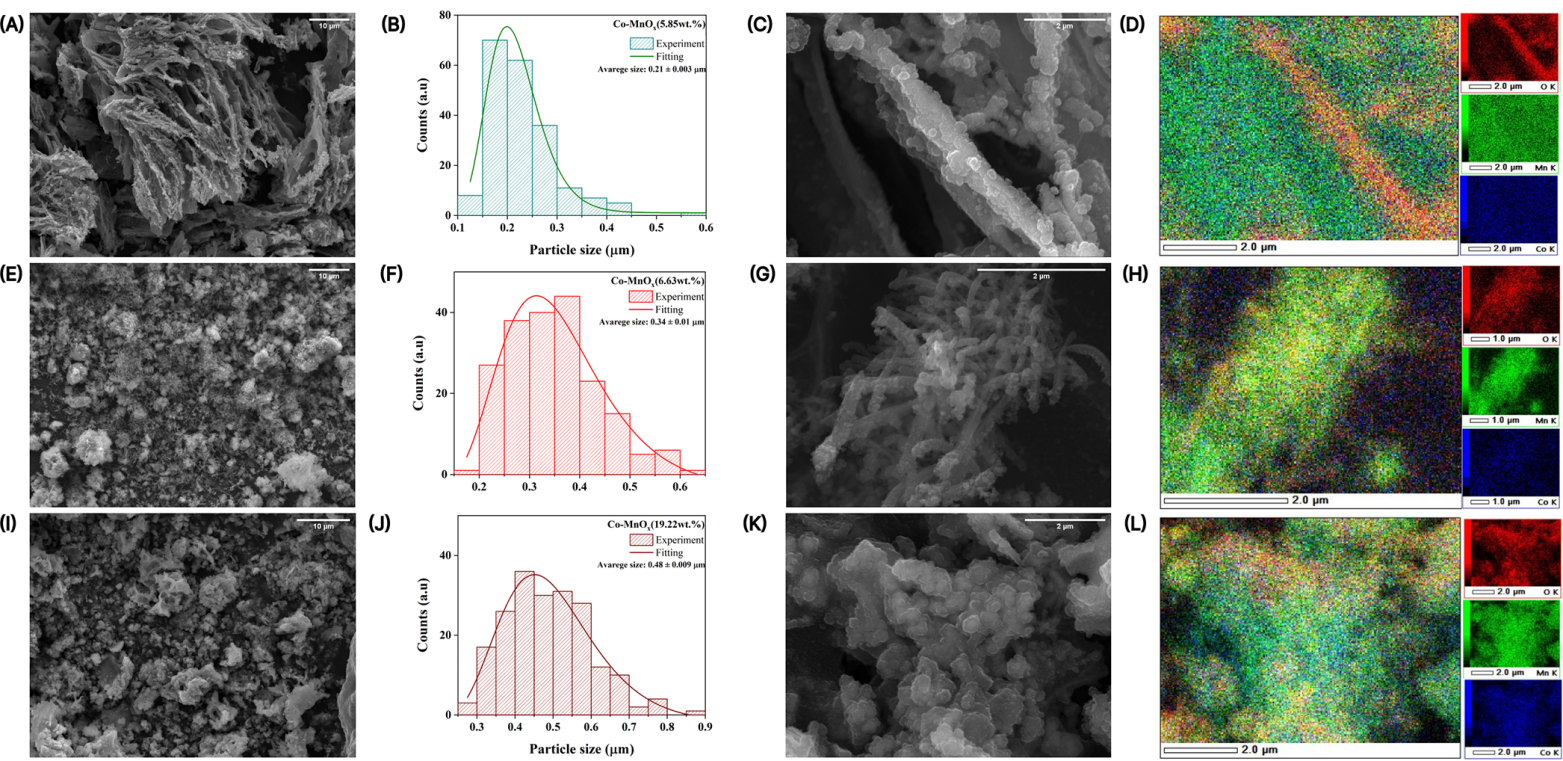
SEM micrographs, cobalt particle size distribution histograms, and EDS elemental mappings of Co-MnO*_x_* samples with different cobalt loadings. (A–D): Co-MnO*_x_* (5.85 wt %); (E–H): Co-MnO*_x_* (6.63 wt %); (I–L): Co-MnO*_x_* (19.22 wt %).

The 6.63 wt % Co content (Figure 2E–H) leads to more surface decoration and agglomerated clusters with a broader size distribution of Co particles (average ≈ 0.34 μm). The high-resolution SEM (Figure 2G) reveals denser cobalt coverage and early signs of particle coalescence, while EDS mapping (Figure 2H) shows a broader cobalt signal, though still evenly distributed. These features match the TEM results (Figure 1D–F), where Co-rich nanoparticles cluster more frequently along the wires. The slight over-incorporation detected by ICP (actual 6.63 wt % vs nominal 5%) suggests an enhanced affinity of the MnO_*x*_ surface for cobalt at this intermediate loading, although some aggregation begins to emerge.

Morphological changes are observed for the 19.22 wt % Co-doped MnO_*x*_ nanowires (Figure 2I–K) with an even wider distribution of Co particles (average ≈ 0.48 μm) (Figure 2J). The SEM micrographs show surface occlusion with large spherical aggregates that obscure the underlying wire network (Figure 2K). EDS maps (Figure 2L) confirm a dominant cobalt signal, with a dense, overlapping distribution across the sample. These observations are in agreement with the TEM images (Figure 1G–I), which display bulky Co-rich domains and heavy surface coverage. The ICP value close to the nominal 20% indicates that nearly all of the added cobalt was deposited, thus exceeding the anchoring capacity of the MnO_*x*_ surface and promoting uncontrolled growth and agglomeration.

The surface chemical states and corresponding relative contributions of the Co-MnO_*x*_ electrode materials were examined using XPS (Figure 3 and Table 1). The Co 2p core-level spectra (Figure 3A,D,G) are characteristic of Co^2+^ species, as evidenced by the Co 2p_3/2_–Co 2p_1/2_ spin–orbit doublet and the presence of intense shakeup satellite peaks (Sat), with no features attributable to Co^3+^. This indicates that cobalt remains predominantly in the +2 oxidation state across all compositions.^5,20^ As the Co content increases, a slight shift to lower binding energies is observed for both Co 2p_3/2_ and Co 2p_1/2_. The binding energy (BE) shifts observed in the Co 2p spectra across the Co-MnO_*x*_ samples can be interpreted based on changes in the local environment of Co^2+^ species. The Co 2p_3/2_ peak appears at 781.6 eV in the 5.85 wt % electrode material, increases slightly to 781.9 eV at 6.63 wt % sample, and then decreases to 780.9 eV in the 19.22 wt % material. The initial increase in BE at intermediate Co content may be attributed to stronger Co–O–Mn interactions, where the electron-withdrawing effect of the Mn environment slightly increases the effective positive charge on Co^2+^, thus shifting the BE to higher values. The relative percentages of the individual oxidation-state contributions were determined from the fitted peak areas and are summarized in Table 1.

**Figure 3:**
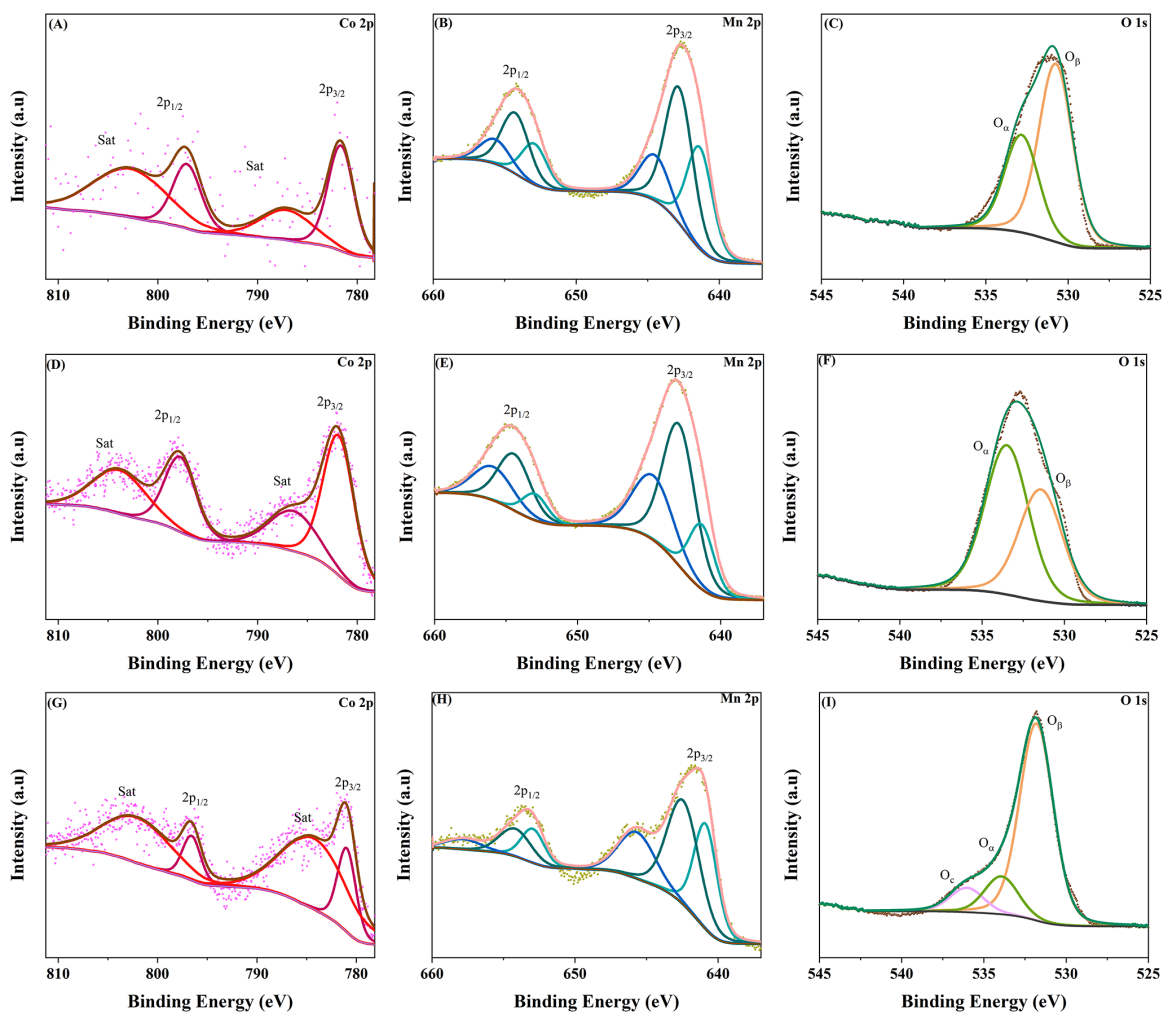
XPS spectra of Co-MnO*_x_* samples with different Co loadings: Co-MnO*_x_* (5.85 wt %)—(A) Co 2p, (B) Mn 2p, (C) O 1s; Co-MnO*_x_* (6.63 wt %)—(D) Co 2p, (E) Mn 2p, (F) O 1s; Co-MnO*_x_* (19.22 wt %)—(G) Co 2p, (H) Mn 2p, (I) O 1s.

**Table 1:** XPS Results for Mn 2p, O 1s, and Co 2p Regions for Co-MnO*_x_* (5.85 wt %), Co-MnO*_x_* (6.63 wt %), and Co-MnO*_x_* (19.22 wt %)^a^

	BE Mn 2p (eV)						BE O 1s (eV)				BE Co 2p (eV)			
Sample	Mn 2p_1/2_			Mn 2p_3/2_			O_c_	O_α_	O_β_	*R*	Sat	Co 2p_1/2_	Sat	Co 2p_3/2_
Co-MnO_x_(5.85 wt %)	655.7 (6.61%)	654.3 (15.01%)	652.9 (9.36%)	644.5 (12.25%)	642.8 (33.5%)	641.4 (23.27%)	-	532.8 (35.41%)	530.8 (64.59%)	0.35	802.8	797.1 (40%)	787.1	781.6 (60%)
Co-MnO_x_(6.63 wt %)	655.9 (10.66%)	654.4 (14.54%)	653.0 (5.25%)	644.7 (20.42%)	643.0 (36.34%)	641.3 (12.78%)	-	533.5 (57.21%)	531.4 (42.79%)	0.57	803.8	797.8 (35%)	786.4	781.9 (65%)
Co-MnO_x_(19.22 wt %)	657.8 (3.93%)	654.2 (9.33%)	652.9 (8.33%)	645.7 (17.8%)	642.4 (34.93%)	640.8 (25.69%)	536.1 (9.41%)	533.9 (14.6%)	531.8 (75.99%)	0.16	802.1	796.6 (34.66%)	784.2	780.9 (65.34%)

a *R*: O_α_/(O_α_ + O_β_).

The O 1s spectra (Figure 3C,F,I) were deconvoluted into contributions from surface-adsorbed oxygen (located at MnO_*x*_ surface oxygen vacancies, O_α_), the lattice oxygen (O_β_),^5,21,22^ and in the case of the 19.22 wt % sample, an additional component (O_c_) attributed to chemisorbed species.^11,23,24^ The main lattice oxygen peak (O_β_) appears at around 530.7–531.8 eV, while O_α_ appears at 532.8–533.9 eV. Notably, the relative intensity ratio *R*, O_α_/(O_α_ + O_β_), increases from 0.35 (5.85 wt %) to 0.57 (6.63 wt %), indicating a higher proportion of lattice oxygen in the intermediate Co content electrode material. The appearance of the O_c_ peak at 536.03 eV in this higher Co-loaded sample supports the presence of additional surface species, such as −OH groups or adsorbed oxygen.^11,25^ For the 19.22 wt % Co-doped MnO_*x*_ nanowires, the *R* value decreases to 0.16, indicating a lower relative fraction of defect-related oxygen compared to lattice oxygen. This suggests that excessive cobalt loading may lead to surface saturation or restructuring, stabilizing the oxide lattice and reducing the concentration of oxygen vacancies.^26^

The Mn 2p core-level spectra (Figure 3B,E,H) reveal the coexistence of Mn^2+^, Mn^3+^ and Mn^4+^ oxidation states, whose relative surface contributions were quantified by peak deconvolution and area analysis.^11^ In Figure 3B (Co-MnO_*x*_ 5.85 wt %), the Mn 2p_3/2_ region displays a dominant peak component centered around 642.8 eV, related to Mn^3+^, then a smaller shoulder at 644.5 eV (Mn^4+^), and a component at 641.4 eV, typical of Mn^2+^. At this low cobalt content, the material has a mixed-valence Mn environment with Mn^3+^ as the prevailing oxidation state. For Co-MnO_*x*_(6.63 wt %), Figure 3E, there is an increase in the relative intensity of the Mn^4+^ component, which now becomes more pronounced with a peak shift to 644.7 eV, while the Mn^3+^ peak remains strong at 643 eV. The Mn^2+^ contribution, at 641.3 eV, is less intense. In Figure 3H (Co-MnO_*x*_ 19.22 wt %), the Mn 2p_3/2_ envelope becomes broader, and the Mn^2+^ component gains relative intensity, centered around 640.8 eV, suggesting a partial reduction of manganese. Although Mn^3+^ and Mn^4+^ species are still present, the increased proportion of Mn^2+^ indicates a shift toward lower oxidation states at high cobalt loading. Therefore, the Mn 2p_3/2_ spectra reveal a nonmonotonic trend: an initial increase in Mn^4+^ content at intermediate Co levels, followed by a partial reduction to Mn^2+^ at higher Co concentrations. This shows the role of cobalt in modulating the redox environment of the manganese within the Co-MnO_*x*_ matrix.

The alterations in the surface chemistry and oxidation states of manganese upon increasing cobalt doping are critical for understanding the electrochemical properties of the Co-MnO_*x*_ nanomaterials. For instance, the notable decrease in the spectral intensity corresponding to oxygen vacancies on the surface when comparing Co-MnO_*x*_(6.63 wt %) to Co-MnO_*x*_(19.22 wt %), Figure 3F,I, occurs concurrently with a significant increase in the Mn^2+^ detected for Co-MnO_*x*_(19.22 wt %), indicating a shift in the average oxidation state of manganese. This phenomenon can be attributed to the inherent charge compensation mechanisms of the system upon the incorporation of higher concentrations of cobalt. When lower-valent cobalt ions (Co^2+^) substitute into the MnO_*x*_ lattice, the charge deficit can be balanced either by the formation of oxygen vacancies or by the reduction of neighboring manganese ions. The results suggest that at higher cobalt doping levels (19.22 wt %), the material preferentially compensates for the charge imbalance through the reduction of manganese to Mn^2+^, rather than solely relying on the generation or retention of oxygen vacancies. This implies that the increasing cobalt content might influence lattice stability or local electronic environments, thereby favoring internal cation reduction as the predominant charge compensation pathway. Such an interplay between oxygen vacancies and manganese oxidation states has been previously noted in various doped manganese oxide systems, highlighting their critical role in dictating material properties and performance, as seen in studies exploring doping effects on charge storage in MnO_*x*_.^27^

The ferromagnetic resonance spectra are presented in Figure 4. The spectra were fitted using combinations of derivative Lorentzian functions, the Tsay et al.^28^ and Griscom^29^ models, and a sigmoidal baseline in cases of additional microwave absorption.^30^ Two main resonances were detected in all samples: a signal centered at ∼3510 G (*g* ≈ 2.0), attributed to paramagnetic centers related to oxygen vacancies in the MnO_2_ matrix. A second signal dependent on Co concentration, associated with the FMR of metallic Co nanoparticles.

**Figure 4:**
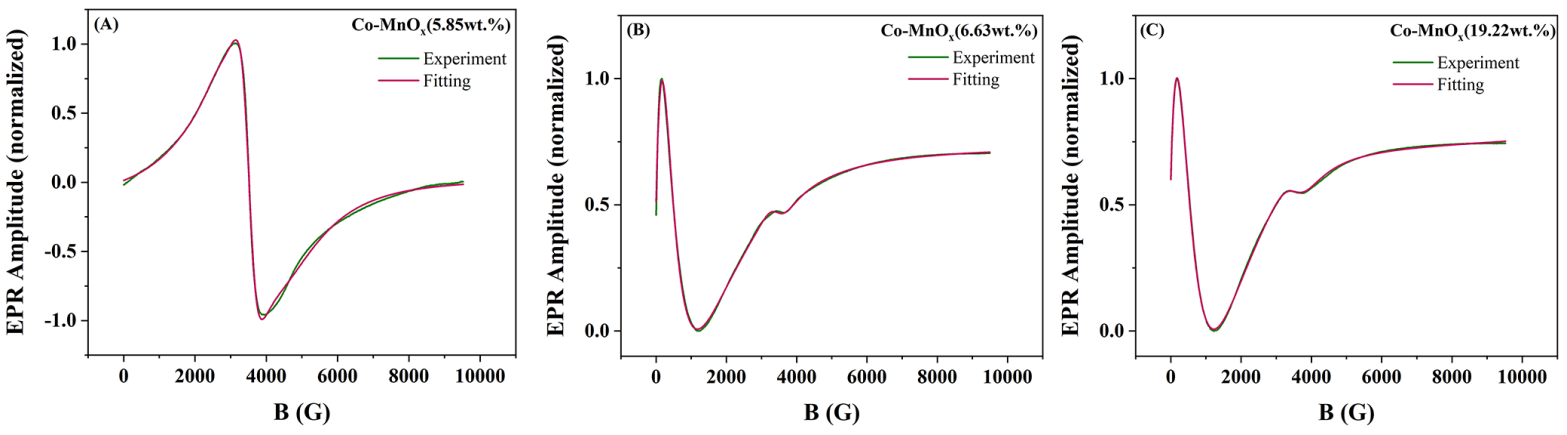
Ferromagnetic resonance spectra of Co-MnO*_x_* (5.85 wt %) (A), Co-MnO*_x_* (6.63 wt %) (B), and Co-MnO*_x_* (19.22 wt %) (C).

For Co-MnO_*x*_(5.85 wt %) sample (Figure 4A) both signals appear near 3500 G but with different line widths (1000 G for FMR and 477 G for EPR). With increasing Co content (6.63 and 19.22 wt %) (Figure 4 B,C), the relative intensity of the FMR signal increases, requiring the inclusion of the sigmoidal baseline in the fits, consistent with microwave absorption by metallic agglomerates.

These results suggest that increasing the Co concentration promotes the formation of metallic agglomerates exhibiting low-field FMR, a behavior commonly observed in nanoparticulate Co systems. This interpretation is consistent with the structural and chemical analyses: TEM and SEM reveal that higher cobalt contents lead to more pronounced particle aggregation and a changes on morphology.

### 3.2 Electrochemical Performance

The electrochemical performance of the electrode materials is presented in Figure 4A–D, with cyclic voltammetry (CV) curves for pure MnO_*x*_ and cobalt-doped MnO_*x*_ with Co contents of 5.85, 6.63, and 19.22 wt %. The measurements were performed in a 2 M KOH aqueous electrolyte at scan rates of 5, 10, 20, 40, and 80 mV·s^–1^ within a potential window from −0.2 to 0.5 V.

The CV curves shows that pure MnO_*x*_ nanowires displays characteristic redox peaks,^14^ while Co-doped samples exhibit changes in both peak intensity and peak position, reflecting altered electrochemical dynamics. Cobalt doping modifies the structure and electronic properties of MnO_*x*_, enhancing redox behavior. With increasing scan rates, all samples show intensified redox signals, indicating kinetic limitations at the electrode/electrolyte interface.^31^ Notably, higher Co concentrations lead to an increase in peak currents, suggesting improved electrical conductivity and charge storage capacity.

The electrochemical behavior of Co-MnO_*x*_ nanomaterials can be correlated with the evolution of surface chemistry and Mn oxidation states observed via XPS (Figure 4B–D). The CV curves have a nonlinear dependence of charge storage performance on Co doping level. In particular, 6.63 wt % Co-MnO_*x*_ shows well-defined CV redox peaks, indicating optimized electrochemical activity at this cobalt doping level. This behavior agrees with XPS results, which reveal its highest relative Mn^4+^ content and largest O_α_/(O_α_ + O_β_) ratio, reflecting a favorable balance of surface oxygen species and high-valent manganese centers.

The redox activity in alkaline electrolytes such as 2 M KOH is primarily governed by the Mn^3+^/Mn^4+^ and Mn^2+^/Mn^3+^ redox couples. At low cobalt content (5.85 wt %), the Mn environment is dominated by Mn^3+^, with some contribution from Mn^2+^ and Mn^4+^. Upon increasing the Co doping to 6.63 wt %, there is a significant increase in Mn^4+^ content as evidenced by the Mn 2p_3/2_ XPS spectra, which shifts toward higher binding energies and reflects a higher oxidation state. This oxidation shift enhances redox kinetics through a greater number of accessible Mn^3+^/Mn^4+^ transitions, which are known to contribute strongly to pseudocapacitance in alkaline media. Moreover, the higher O_α_/(O_α_ + O_β_) ratio at this doping level suggests more surface-active oxygen species, which facilitate faster electron/proton exchange.

Interestingly, when the Co content reaches 19.22 wt %, a reverse trend is observed. The Mn^2+^ contribution becomes dominant, and the Mn 2p envelope broadens, indicating a partial reduction of Mn. At the same time, the O_α_ contribution decreases while an additional O_c_ component appears, attributed to chemisorbed −OH or O^–^ species. These changes reflect a shift in charge compensation mechanisms: at high Co levels, instead of forming more oxygen vacancies, the system compensates via the reduction of Mn ions. This shift toward lower Mn oxidation states reduces the number of high-energy Mn^3+^/Mn^4+^ redox sites, leading to a decline in redox peak current. These findings are consistent with prior reports showing that excessive Co^2+^ doping can disrupt MnO_*x*_ lattice structure and hinder electron transfer pathways by promoting Mn^2+^ formation and suppressing redox-active sites.^32^

These transitions occur at different potentials, contributing to the observed redox peaks in CV and governing the overall capacitive behavior. The intermediate Co doping (6.63 wt %) appears to optimize this redox framework by maximizing Mn^4+^ content and enhancing surface reactivity, whereas excessive doping (19.22 wt %) leads to charge imbalance and over-reduction of Mn, resulting in performance decline.

The Randles–Ševčík plots, Figure 5E, show the linear relationship between the peak current (*I*_p_) and the square root of the scan rate (ν^1/2^). This analysis elucidates the diffusion characteristics and the electrochemical reaction mechanisms of the samples. All plots exhibit strong linearity, with *R* ^2^ values exceeding 0.99, indicating that the peak current is directly proportional to ν^1/2^. This behavior is characteristic of diffusion-controlled processes, suggesting that ion transport through the electrolyte and electrode interface plays a key role in the charge storage mechanism.^33^

**Figure 5:**
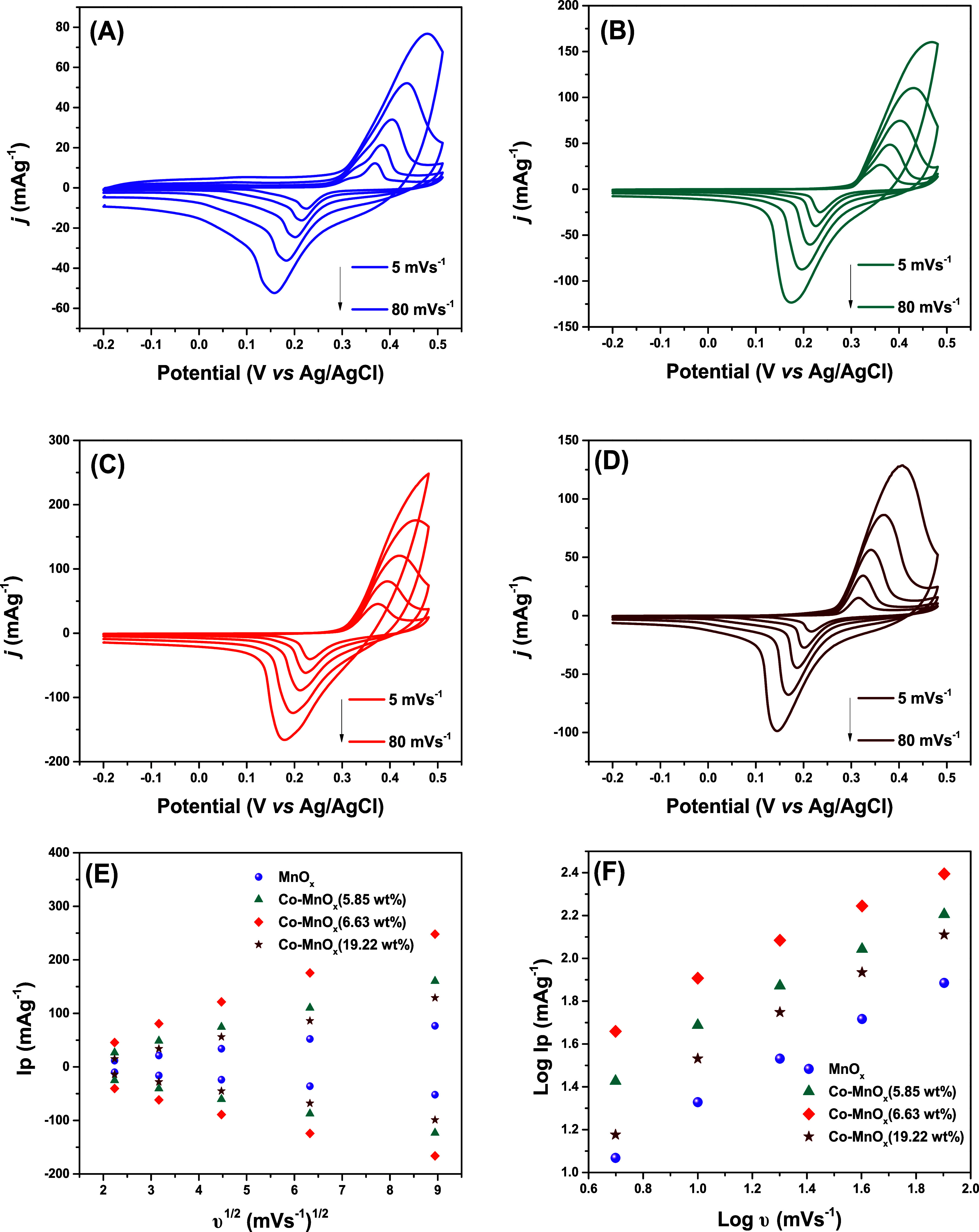
Cyclic voltammetry analysis, MnO*_x_* (A), Co-MnO*_x_* (5.85 wt %) (B), Co-MnO*_x_* (6.63 wt %) (C) and Co-MnO*_x_* (19.22 wt %) (D). Peak current vs square root of scan rate (E), log of peak current vs log of scan rate (F).

Furthermore, it was possible to calculate the specific capacitance (*C*_s_) of the samples using the CV curves and applying eq 2.^34^ The calculations were conducted for different scanning speeds, and the results obtained were organized and detailed in Table 2.

2 $$C_{s}=\frac{1}{2\times m\times \nu \times \Delta V}\times \int I\left(V\right)dV$$

**Table 2:** Specific Capacitances Calculated from CV Curves for MnO*_x_*, Co-MnO*_x_* (5.85 wt %), Co-MnO*_x_* (6.63 wt %), and Co-MnO*_x_* (19.22 wt %)^a^

	Specific capacitance (F·g^–1^)			
ν	MnO_*x*_	Co-MnO_*x*_(5.85 wt %)	Co-MnO_*x*_(6.63 wt %)	Co-MnO_*x*_(19.22 wt %)
5	273.81	627.02	460.69	439.67
10	279.76	636.22	441.78	483.30
20	255.95	571.16	355.51	457.24
40	224.70	469.16	243.83	411.69
80	168.90	306.42	142.49	351.79

a *ν* = scan rate (mVs^–1^).

Where *C*_s_ corresponds to the specific capacitance (F·g^–1^), *m* the active mass of the electrode (g), ν the scanning speed in (mV·s^–1^), *V* the potential window (V) and *I*(v) the resulting current (A). The similar *C*_s_ values observed at scan rates of 5 and 10 mV s^–1^ for both pure MnO_*x*_ and Co-doped MnO_*x*_ nanowires indicate that, in this low scan-rate regime, electrochemical charge storage is not kinetically limited. For pristine MnO_*x*_, the slow potential sweep allows sufficient time for electrolyte ions to access the available surface and near-surface redox sites, resulting in nearly complete charge utilization at both scan rates. In the Co-doped samples, this behavior is further reinforced by the presence of cobalt species, which enhance electronic conductivity and facilitate faster charge-transfer processes. For all samples analyzed, Table 2. As the scan rate increases, the time available for ion diffusion and effective electrode polarization decreases, leading to a reduction in *C*_s_.^8^

To evaluate the energy storage mechanism for Co-MnO_*x*_(6.63 wt %), an analysis was conducted based on the application of the power law, eq 3.^35^ This method enables to investigate the contributions of multiple processes, including charge storage capacity, ion transport kinetics, and electron transfer. It also allows to identify the key characteristics influencing energy storage efficiency, thereby determining whether the samples exhibit battery-type or capacitive behavior.^36^

3 $$i=a\nu^{b}↔\log\left(i\right)=b\times \log\left(\nu \right)\times \log\left(a\right)$$

where, *i* corresponds to the current density (A·g^–1^) and ν the variation in scanning speed (mV·s^–1^) and *b* acts as a control parameter. From eq 3, the value of the parameter *b* can be estimated (Table 3). When *b* ≥ 0.5, the material exhibits battery-like behavior. If *b* approaches 1.0 or exceeds it, the charge storage process is predominantly capacitive. Conversely, values of *b* between 0.5 and 1.0 indicate a combination of battery-type and capacitive energy storage mechanisms. The average *b* value for Co-MnO_*x*_(6.63 wt %) was 0.62, suggesting that this electrode material combine redox and capacitive processes in energy storage.

**Table 3:** Power Law Coefficients for Co-MnO*_x_* (6.63 wt %)^a^

	***b***	
ν	Anodic	Cathodic
5	0.6	0.8
10	0.7	0.5
20	0.5	0.6
40	0.6	0.6
80	0.6	0.7

a ν = scan rate (mVs^–1^).

The contributions of the capacitive and diffusional currents on Co-MnO_*x*_(6.63 wt %) were understood by applying eqs 4–5.^37,38^ Where *i* corresponds to current as a function of potential, ν is the scanning speed, *k*_1_ is a constant related to fast storage processes and *k*_2_ is a constant related to slow diffusion processes:^39^

4 $$i\left(V\right)=k_{1}\nu +k_{2}\nu^{1/2}$$

5 $$i\left(V\right)/\nu^{1/2}=k_{1}\nu^{1/2}+k_{2}$$

The *i*(*v*)/ν^1/2^ versus ν^1/2^ plot was used to estimate the values of *k*_1_ and *k*_2_, obtained from the slope and intercept, respectively, as illustrated in Figure 6A.^40^ These parameters are essential for separating the capacitive and diffusion-controlled contributions in the electrochemical behavior. For Co-MnO_*x*_(6.63 wt %) at a scan rate of 5 mV·s^–1^, the capacitive and diffusion-controlled contributions were 15.22% and 84.78%, respectively, as shown in Figure 6A. This result indicates a dominance of diffusion-controlled currents, suggesting a significant role of pseudocapacitive mechanisms, possibly due to redox reactions involving cobalt and manganese oxide.

**Figure 6:**
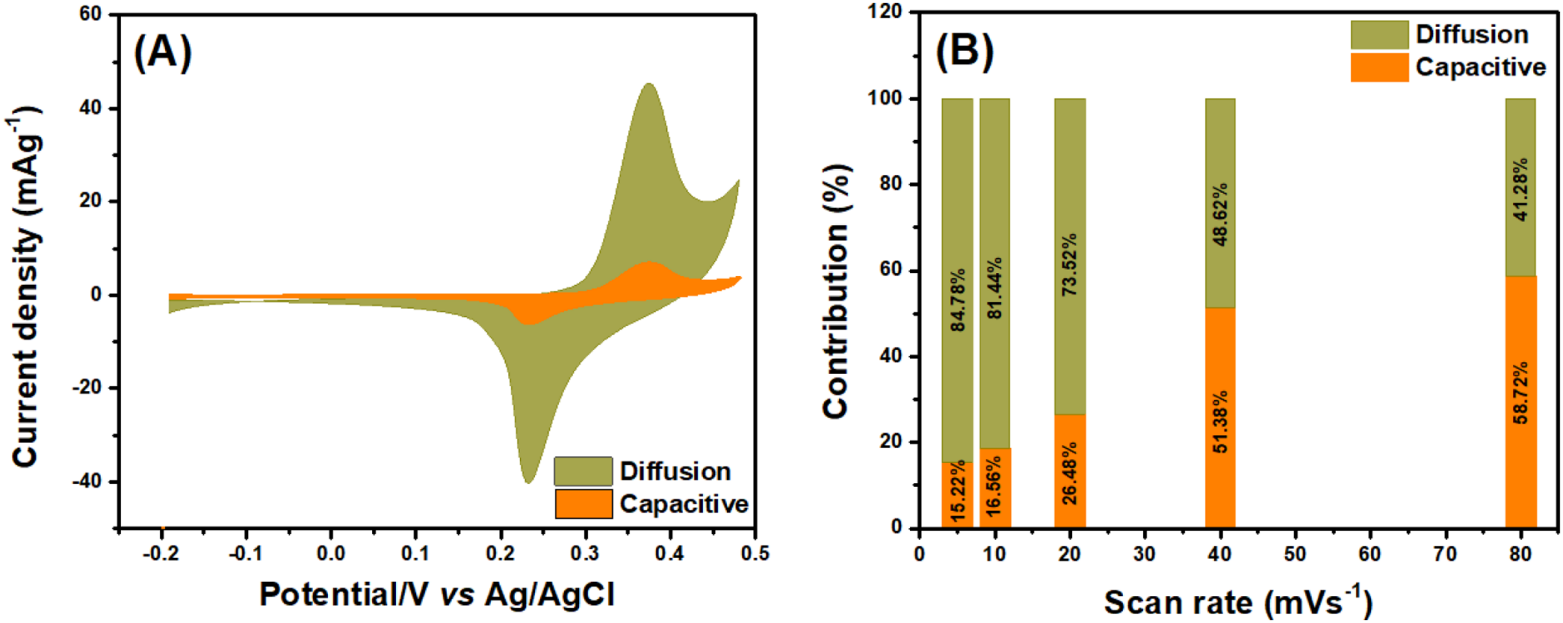
(A) CV of the capacitive/diffusion-controlled contributions and (B) percentage of the diffusion and capacitive contributions at different scan rates, for Co-MnO*_x_* at 5 mV·s^– 1^, respectively.

The ratio of diffusion-controlled to capacitive contributions evolves significantly as the scan rate increases from 5 to 80 mV·s^–1^, Figure 6B. At low scan rates, diffusion-limited Faradaic reactions dominate, but as the sweep rate increases, redox species cannot diffuse fast enough, and the response shifts toward electric double-layer charging. Thus, higher scan rates enable faster charge storage suitable for high-power applications, although the reduced Faradaic contribution lowers the overall energy density. This aligns with findings in Co-doped manganese oxide synthesized using sol–gel method, authors observed that capacitive response increases with scan rate, while diffusion-controlled current drops, since fast potential sweeps limit the time available for redox reactions to occur.^41^

To accurately evaluate the charge storage behavior of pure MnO_*x*_ and Co-doped MnO_*x*_ electrode materials we investigated using the galvanostatic charge and discharge technique (GCD), Figure 7. The experiments were conducted at current densities of 1, 2, 5, 10, and 20 A·g^–1^, enabling an analysis of the performance of the samples, and in a potential window that varied from 0.0 to 0.42 V.

**Figure 7:**
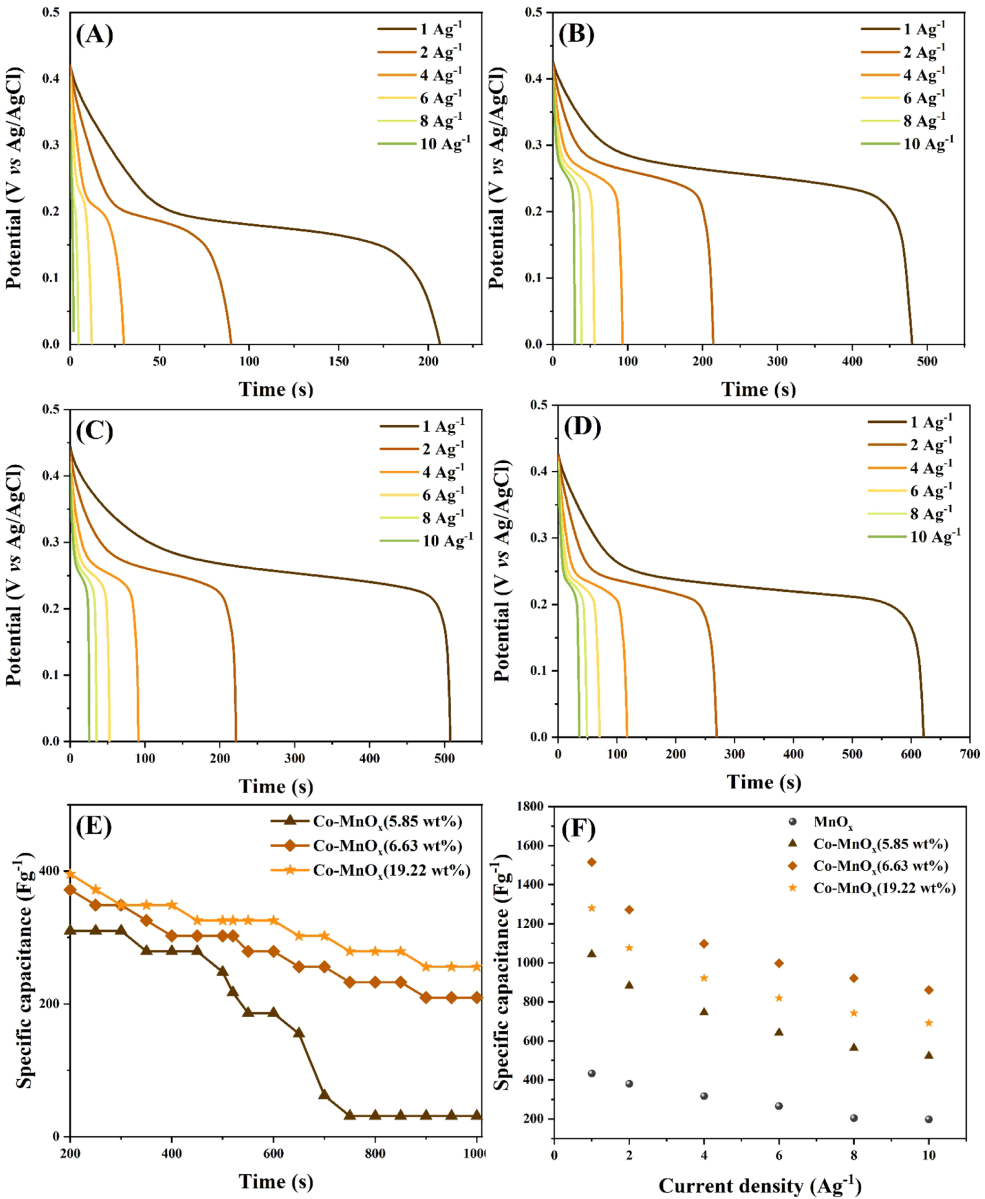
Discharge curves of the GCD technique, MnO*_x_* (A), Co-MnO*_x_* (5.85 wt %) (B), Co-MnO*_x_* (6.63 wt %) (C) and Co-MnO*_x_* (19.22 wt %) (D). Stability over 1000 GCD cycles at 10 A·g^–1^(E) specific capacitance vs current density (F).

The specific capacitances were determined from the discharge curves of the electrode materials, using eq 6:^42^

6 $$C_{s}=\frac{I\times \Delta T}{\Delta V\times m}$$

Where *C*_s_ represents the specific capacitance (F·g^–1^), *I* is the current (A), Δ*T* is the discharge time (s), Δ*V* is the potential variation (V), and *m* is the active mass of the material (g).

The Coulombic efficiency was determined using eq 7:^43^

7 $$\eta \%=\frac{T_{d}}{T_{c}}\times 100$$

where η represents the Coulombic efficiency (%), *T*_d_ is the discharge time, and *T*_c_ is the charge time.

The GCD results summarized in Table 4 and Figure 7A–D show enhancement in the electrochemical performance of MnO_*x*_ nanowires upon cobalt incorporation. Bare MnO_*x*_ nanowires exhibits a moderate *C*_s_ of 432.65 F·g^–1^at 1 A·g^–1^, which decreases steadily with increasing current density, reflecting limited rate capability. In contrast, all Co-doped samples deliver higher capacitance values across the current density range, demonstrating improved charge storage and kinetics. Among the doped electrodes, Co-MnO_*x*_(6.63 wt %) shows the highest specific capacitance, reaching 1468.65 F·g^–1^ at 1 A·g^–1^, indicating an optimal balance between enhanced electronic conductivity and accessible redox-active sites. Although its capacitance decreases at higher current densities, it remains superior to the pristine MnO_*x*_ and other doped samples, confirming improved rate performance. The Co-MnO_*x*_(5.85 wt %) and Co-MnO_*x*_(19.22 wt %) electrode materials also exhibit higher *C*_s_ compared to MnO_*x*_, but their lower values suggest either insufficient cobalt content or excessive surface coverage and particle agglomeration, respectively.

**Table 4:** Specific Capacitance from the Discharge Curves and Coulombic Efficiency for MnO*_x_*, Co-MnO*_x_* (5.85 wt %), Co-MnO*_x_* (6.63 wt %), and Co-MnO*_x_* (19.22 wt %)^a^

	MnO_*x*_		Co-MnO_*x*_(5.85 wt %)		Co-MnO_*x*_(6.63 wt %)		Co-MnO_*x*_(19.22 wt %)	
Current density (Ag^–1^)	*C*_s_	η	*C*_s_	η	*C*_s_	η	*C*_s_	η
1	432.65	84.46	1043.18	84.53	1468.65	84.52	1279.73	87.36
2	379.59	95.87	881.81	96.41	1272.09	93.67	1076.95	94.08
4	316.32	96.87	745.45	97.62	1097.67	96.84	921.56	96.72
6	265.30	100	640.91	98.24	997.67	96.36	819.29	97.26
8	204.08	100	563.64	97.50	920.93	97.30	742.28	96.07
10	198.06	100	522.73	100	860.47	96.30	691.6	100

a *C*_s_: Specific capacitance (F·g^–1^) and η: Coulombic efficiency (%)

The differences in specific capacitance values and trends obtained from CV (Table 2) and GCD measurements arise from the distinct nature of the techniques, as CV can overestimate capacitance due to transient currents and overlapping faradaic and capacitive contributions. In contrast, GCD directly measures charge–discharge behavior under constant current, providing a more realistic evaluation of charge-storage capability. Therefore, GCD-derived *C*_s_ values are considered more reliable for comparing the electrochemical performance of the electrodes.

In Table 4, all samples exhibit high Coulombic efficiency (η), indicating charge/discharge reversibility. The sample with 6.63 wt % Co maintains high η values above 84% across all current densities, confirming efficient redox processes even under high charge/discharge rates.

Table 5 compares the electrochemical performance of various Mn-based electrode materials reported in the literature. The Co-MnO_*x*_(6.63 wt %) synthesized in this work exhibits the highest specific capacitance (1468.65 F·g^–1^ at 1 A·g^–1^) among the listed materials, outperforming other compositions such as Ni–Co–Mn (1360 F·g^–1^) and Zn-doped MnO_*x*_ (1082.2 F·g^–1^). These results show the beneficial role of moderate Co incorporation in enhancing charge storage performance, due to improved electronic conductivity and synergistic redox activity between Co and Mn species. The Co-MnO_*x*_(6.63 wt %) electrode achieved a much higher specific capacitance even with a lower electrolyte concentration (2 M KOH), suggesting that the superior material properties such as optimized morphology, surface area, and redox synergy play a more dominant role than electrolyte concentration alone in enhancing charge storage performance.

**Table 5:** Electrochemical Performance of Serveral Mn Based Materials

Samples	Electrolytic	Specific capacitance (F·g^–1^)	Ref
Co-MnO_*x*_(6.63 wt %)	2 M KOH	1468.65 (1 A·g^–1^)	This work
CoMn_2_O_4_	1 M KOH	323 (0.5 A·g^–1^)	^13^
Co_2.16_Mn_0.84_(PO_4_)_2_	1 M KOH	571 (2.2 A·g^–1^)	^15^
P-Co_2_MnO_4–*x*_	3 M KOH	838 (1 A·g^–1^)	^44^
Ni–Co–Mn	3 M KOH	1360 (1 A·g^–1^)	^45^
Mn–O–C composite	1 M Na_2_SO_4_	550 (1 A·g^–1^)	^46^
CoMn_2_O_4_	6 M KOH	614.8 (1 A·g^–1^)	^12^
Co-doped ZnMn_2_O_4_	1 M KOH	1196 (1 A·g^–1)^	^47^
Co-doped MnO_2_	1 M MgSO_4_	527 (1 A·g^–1^)	^48^
Zn doped MnO_*x*_	2 M KOH	1082.2 (1 A·g^–1^)	^23^

Cycling stability is another key feature of energy storage devices. Figure 7E shows the specific capacitance over 1000 charge–discharge cycles at a high current density of 10 A·g^–1^ for the Co-MnO_*x*_ electrode materials. Co-MnO_*x*_(6.63 wt %) and Co-MnO_*x*_(19.22 wt %) demonstrate superior cycling stability compared to both MnO_*x*_ and the lower Co content sample (5.85 wt %). Notably, Co-MnO_*x*_(6.63 wt %) maintains high capacitance retention for a lower Co content, which can be attributed to the improved conductivity and enhanced structural stability introduced by optimal Co incorporation. In contrast, bare MnO_*x*_ nanowires suffers a sharp decline in capacitance, suggesting poor long-term durability under high-rate operation. Figure 7F presents the specific capacitance as a function of current density. As the current density increases, a decline in *C*_s_ is observed for all samples, typical of diffusion limitations at high charge/discharge rates.^31^ Across all current densities (1–10 A·g^–1^), Co-doped samples outperform the undoped MnO_*x*_, with the 6.63 wt % Co-MnO_*x*_ composition exhibiting the highest specific capacitance at lower current densities.

Electrochemical Impedance Spectroscopy (EIS) tests were conducted in parallel with the cycling stability study over a frequency range 0.01 to 10^5^ Hz with an amplitude of 10 mV^–1^. These measurements were performed before and after 500 and 1000 charge–discharge cycles to evaluate the electrochemical behavior and long-term stability of the materials during the GCD process. In all samples, the formation of a semicircle at low frequencies was observed, from which the associated resistances were determined by extrapolation.^49^ The impedance spectra, presented in Figure 8 as Nyquist plots, reveal distinct behaviors of the system. From these spectra, it was possible to extract data such as Series Resistance (*R*_s_), Charge Transfer Resistance (*R*_ct_), Electrical Double Layer Capacitance (*C*_dl_) and Warburg Resistance/Impedance (*Z*_w_), presented in Table 6. The Nyquist graph is composed of the real part (Z′), represented on the horizontal axis, and the imaginary part (−Z″), displayed on the vertical axis.^42,50,51^

**Figure 8:**
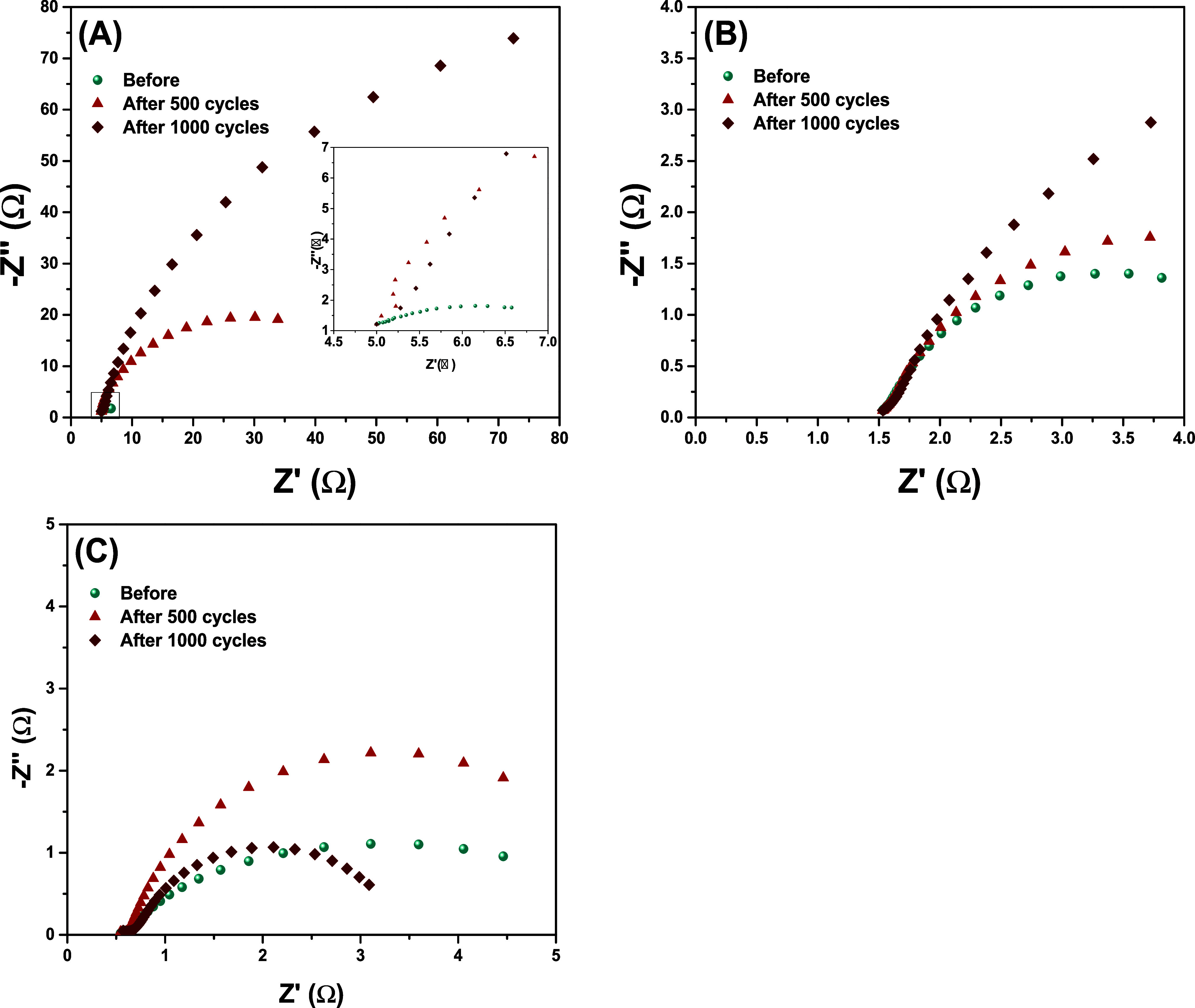
Nyquist plot from the EIS spectra for the samples, Co-MnO*_x_* (5.85 wt %) (A), Co-MnO*_x_* (6.63 wt %) (B) and Co-MnO*_x_* (19.22 wt %) (C).

**Table 6:** Evolution of Electrochemical Impedance Parameters for Co-MnO*_x_* Samples (5.85, 6.63, and 19.22 wt %) before and after 500 and 1000 Charge–Discharge Cycles^a^

	Co-MnO_x_(5.85 wt %)			Co-MnO_x_(6.63 wt %)			Co-MnO_x_(19.22 wt %)		
Cycles	*R*_s_	*R*_ct_	*C*_dl_	*R*_s_	*R*_ct_	*C*_dl_	*R*_s_	*R*_ct_	*C*_dl_
Before cycling	4.98 Ω	2.33 Ω	5.34 μF	1.83 Ω	3.65 Ω	302 μF	0.57 Ω	2,67 Ω	141 μF
After 500 cycles	1.90 Ω	51.57 Ω	0.75 μF	1.28 Ω	9.76 Ω	164 μF	0.57 Ω	1.18 Ω	529 μF
After 1000 cycles	4.63 Ω	201.45 Ω	7.90 μF	1.56 Ω	4.56 Ω	298 μF	0.57 Ω	1.01 Ω	564 μF

a *R*_s_: Series resistance; *R*_ct_: Charge transfer resistance; *C*_dl_: Electrical double-layer capacitance

For the Co-MnO_*x*_(5.85 wt %) sample, the series resistance (*R*_s_) was 4.98 Ω throughout the entire GCD process. The charge transfer resistance (*R*_ct_) values varied from 2.33 Ω before cycling, to 51.57 Ω after 500 cycles, and 201.45 Ω after 1000 cycles. In contrast, the Co-MnO_*x*_(6.63 wt %) sample had an initial *R*_s_ of 1.83 Ω, with *R*_ct_ values of 3.65 Ω before cycling, 9.76 Ω after 500 cycles, and 4.56 Ω after 1000 cycles. Co-MnO_*x*_(19.22 wt %) showed the lowest initial *R*_s_ of 0.57 Ω, with *R*_ct_ values of 2.67 Ω before cycling, 1.18 Ω after 500 cycles, and 1.01 Ω after 1000 cycles. The significant increase in *R*_ct_ for the sample with 5.85% Co indicates a higher resistance to charge transfer over the 1000 charge–discharge cycles, resulting in lower stability of *C*_s_ during the analysis. In contrast, the samples with 6.63% and 19.22% Co exhibited a decrease in *R*_ct_ over the cycles, indicating an improvement in the charge transfer process and, consequently, better retention of specific capacitance during the GCD analysis.

These results indicate an increase in charge transfer resistance over the number of cycles. This may be attributed to changes in the electrode–electrolyte interface during charge and discharge cycles. These changes in charge transfer resistance can impact the efficiency of the supercapacitor, reflecting a material degradation process or accumulation of electrolyte ions in the KOH solution.^52^

The supercapattery has two electrodes: the positive electrode containing the active material, and the negative electrode with activated carbon (AC). To calculate the amount of activated carbon required for this configuration, the mass-to-charge ratio was used, as expressed by eq 8:^31^

8 $$\frac{m^{+}}{m^{-}}=\frac{Q^{+}}{Q^{-}}=\frac{m\times C_{s}\times \Delta V}{m\times C_{s}\times \Delta V}$$

*m* represents the mass of the electrode (mg), *C*_s_ is the specific capacitance, and Δ*V* refers to the potential difference observed for both the activated carbon and the active material. This allows for the precise determination of the amount of activated carbon required, optimizing the material ratio and ensuring balance between energy storage capacity and operational efficiency of the supercapattery.

The cyclic voltammetry (CV) curves of AC in the potential range of −1.2 to −0.3 V and Co-MnO_*x*_(6.63 wt %) in the range of −0.2 to 0.5 V, both measured at a scan rate of 5 mVs^–1^ is featured in Figure 9A. While AC displays a nearly rectangular CV profile, characteristic of electric double-layer capacitance (EDLC), the Co-MnO_*x*_(6.63 wt %) curve exhibits distinct redox peaks, confirming the pseudocapacitive nature of the material due to Faradaic redox reactions involving Mn and Co species.

**Figure 9:**
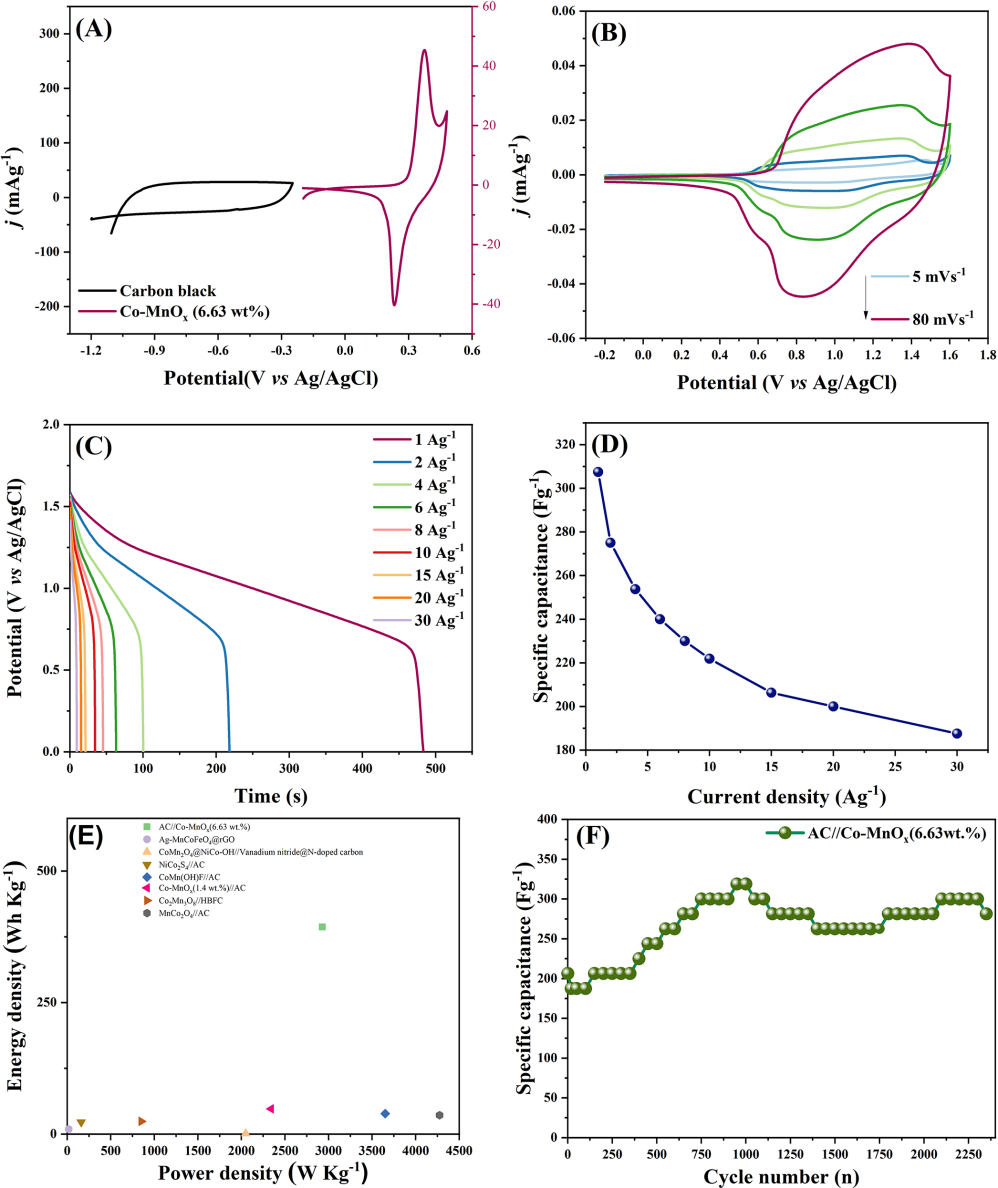
Cyclic voltammetry curves for activated carbon and Co-MnO*_x_* (A), cyclic voltammetry for the supercapattery Co-MnO*_x_* (6.63 wt %)//AC (B), discharge curves at different current densities (C), specific capacitance versus current density (D), Ragone Plot (E) and cycling stability test at 30 A·g^–1^ (F).

The AC//Co-MnO_*x*_(6.63 wt %) supercapattery was analyzed using CV a potential range of 0 to 1.6 V, with scan rates varying between 5 and 200 mVs^–1^, and in a 2 mol L^–1^ KOH electrolyte. Figure 9B shows the device voltammogram, featuring a distorted rectangular shape with mild redox signals, reflecting the combined behavior of AC and Co-MnO_*x*_ electrodes. The nearly symmetric curves across all scan rates indicate good reversibility and stable charge storage, and the preservation of this shape at higher scan rates suggests efficient ion transport and structural stability.^53^

The discharge curves for AC//Co-MnO_*x*_(6.63 wt %) device were obtained at a series of current densities, including 1, 2, 4, 6, 8, 10, 15, 20, and 30 A·g^–1^, Figure 9C. Their nonlinear profiles further confirm the contribution of pseudocapacitance, and the relatively long discharge durations at low current densities reflect its high energy storage capability. Based on these curves, the supercapattery delivers a *C*_s_ of 307.5 F·g^–1^ at 1 A·g^–1^. As shown in Figure 9D, the discharge time shortens with increasing current density due to the limited availability of active sites on the electrode. Even so, the device maintains 187.5 F·g^–1^ at 30 A·g^–1^, demonstrating excellent rate capability.

To evaluate the performance of the device, the energy and power densities were calculated using eqs 9–10, as previously described.^3^

9 $$ED=\frac{1}{2}\times C_{s}{\left(\Delta V\right)}^{2}$$

10 $$PD=3600\times \frac{ED}{\Delta T}$$

*C*_s_ is the specific capacitance (F·g^–1^) obtained in the GCD tests, Δ*V* (V) is the potential range between the cathode and anode. Δ*T* (s) is the time of discharge of the device. All tests were carried out at room temperature.

The specific capacity, energy density (ED) and power density (PD) of the AC//Co-MnO_*x*_(6.63 wt %) supercapattery were calculated, Table 7. The results reveal a typical trend for hybrid systems: *C*_s_ and ED gradually decrease with increasing current density, due to limited ion diffusion and reduced utilization of active sites at higher rates. Despite this, the device retains 61% of its initial *C*_s_ (from 307.5 to 187.5 F·g^–1^) even at 30 A·g^–1^, highlighting its rate capability. To contextualize these results, a comparative analysis with previously reported Co/Mn-based supercapatteries was performed in Table 8 and further illustrated in the Ragone plot shown in Figure 9E. The AC//CoMnO_*x*_(6.63 wt %) device developed in this work exhibits a high energy density of 393.6 Wh·kg^–1^ and a power density of 2928 W·kg^–1^, surpassing the performance of other materials listed. The cycling stability profile of AC//Co-MnO_*x*_(6.63 wt %) at 30 A·g^–1^, as shown in the Figure 9F, demonstrates long-term electrochemical durability. The increase in *C*_s_ after around 2200 cycles is attributed to progressive electrochemical activation of the positive electrode, a behavior commonly reported for transition metal oxide-based hybrid supercapacitors.^44,54^ Prolonged cycling enhances electrode wetting and electrolyte penetration, gradually activating initially inactive redox sites.^55^ XPS results show that the 6.63 wt % Co sample presents the highest Mn^4+^/Mn^3+^ ratio and the largest contribution of surface oxygen species, facilitating the progressive participation of Mn^4+^/Mn^3+^ and Co^2+^/Co^3+^ redox couples. Additionally, oxygen-vacancy-related defects act as reversible charge storage centers, leading to capacity values exceeding the initial performance while maintaining long-term electrochemical stability.^4,56^ The retention of *C*_s_ over extended cycling confirms the structural stability of the electrode material and reversibility of redox reactions, making it a strong candidate for practical energy storage applications. The PD and ED for AC//Co-MnO_*x*_(6.63 wt %) demonstrate not only robust performance, but also indicate that Co-MnO_*x*_, in combination with activated carbon (AC), can be an alternative for applications that require high efficiency in energy storage.

**Table 7:** Electrochemical Performance of AC//Co-MnO*_x_* (6.63 wt %) Supercapattery

	AC//Co-MnO_*x*_(6.63 wt %)		
Current density (Ag^–1^)	*C*_s_ (F·g^–1^)	Energy density Wh·kg^–1^ (ED)	Power density W·kg^–1^ (PD)
1	307.5	393.6	2928
2	275.0	352.0	5786
4	253.7	324.8	11577
6	240.0	307.2	17280
8	230.0	294.4	23040
10	221.9	284.0	29212
15	206.2	264.0	43200
20	200.0	256.0	57600
30	187.5	240.0	86400

**Table 8:** Performance of Co/Mn Based Materials in Supercapattery

Samples	Electrolyte	Energy density Wh·kg^–1^ (ED)	Power density W·kg^–1^ (PD)	Ref
AC//Co-MnO_*x*_(6.63 wt %)	2 M KOH	393.6	2928	This work
Ag-MnCoFeO_4_@rGO	Solid electrolytes	9.28	18	^57^
MnCo_2_O_4_//AC	1 M KOH	36	4274	^58^
CoMn_2_O_4_@NiCo–OH//Vanadium nitride@N-doped carbon	1 M KOH	68.83	2048	^59^
NiCo_2_S_4_//AC	2 M KOH	22.8	160	^60^
CoMn(OH)F//AC	3 M KOH	39	3650	^61^
Co-MnO_*x*_(1.4 wt %)//AC	2 M KOH	48.04	2,339.02	^14^
Co_2_Mn_3_O_8_//HBFC	2 M KOH	24.3	850	^62^

The excellent electrochemical activity of the Co-MnO_*x*_(6.63 wt %) electrode material, both in the three-electrode configuration and when assembled as a supercapattery, can be attributed to its balance between surface defects, mixed-valence manganese, and structural stability. XPS analysis reveals that this material exhibits the highest relative fraction of defect-related oxygen species (O_α_), indicating an increased density of reactive surface sites, together with a favorable Mn^3+^/Mn^4+^ ratio. Moreover, the incorporation of Co^2+^ promotes electronic interactions with the MnO_*x*_ lattice, enhancing redox activity without inducing surface saturation.

## 4 Conclusion

This study demonstrates that cobalt doping through a urea-assisted hydrolysis route enables modulation of MnO_*x*_ nanowire morphology, surface chemistry, and redox activity. The results indicate that an intermediate cobalt content (6.63 wt %) provides an optimal balance between surface oxygen defects, mixed-valence manganese, and structural stability, resulting in enhanced electrochemical activity. Higher cobalt loadings disrupt this balance through surface saturation and changes in manganese oxidation states. Electrochemical testing revealed that this electrode material exhibited the highest specific capacitance, outstanding rate capability, and long-term cycling stability. The AC//Co-MnO_*x*_(6.63 wt %) supercapattery achieved high energy and power densities, surpassing several reported Mn-based systems. These findings establish a structure–property–performance correlation, highlighting the importance of optimizing dopant concentration to maximize electrochemical storage performance. Moreover, the scalable synthesis route and the favorable energy storage characteristics make this electrode material a promising candidate for supercapattery applications.

## Supplementary Material



## Notes

Representative TEM images of Co-MnO_*x*_ samples with different Co loadings analyzed using ImageJ (PDF)
