# Correction to “Tailoring Cobalt Content in MnO_*x*_ Nanowires for Superior Supercapattery Performance”

**DOI:** 10.1021/acsomega.6c02806

**Published:** 2026-04-03

**Authors:** Fernando José Soares Barros, Samuel da Silva Eduardo, Klebson Lucas Pereira Cardozo, Hector Aguilar Vitorino, Carlos Martins Aiube, Mariana Lumi Ichihara Sado, Camila de Lima Ribeiro, Paulo Eduardo Narcizo de Souza, Alysson Martins Almeida Silva, Auro Atsushi Tanaka

**Affiliations:** † Department of Chemistry, 37892 Federal University of Maranhão, Av. dos Portugueses, 1966, São Luís, MA 65080-805, Brazil; ‡ Department of chemistry, 28125 Universidade Federal do Rio de Janeiro, Avenida Athos da Silveira Ramos, n° 149, 21941-909, Rio de Janeiro, RJ 21941-901, Brazil; § Department of Fundamental Chemistry, Institute of Chemistry, University of São Paulo, Av. Prof. Lineu Prestes, 748, São Paulo, SP 05508-000, Brazil; ∥ Institute of Chemistry, 28127 University of Brasília, Campus Universitário Darcy Ribeiro, Asa Norte, Brasília, DF 70910-900, Brazil; ⊥ Department of Mechanical Engineering, University of Brasilia, Campus Universitário Darcy Ribeiro, Asa Norte, Brasília, DF 70910-900, Brazil; # Institute of Physics, University of Brasilia, Campus Universitário Darcy Ribeiro, Asa Norte, Brasília, DF 70910-900, Brazil

We are issuing a correction to amend the name of one of the authors in the original published article. The author’s name was previously incorrect and has now been updated to the correct form. This change is reflected in the authorship of this Correction. The Author Information with this Correction reflects the correction.

Original content:

Hector A. Aguilar Vitorino

Corrected content:

Hector Aguilar Vitorino

