# Supplementary material for: Tailoring Cobalt Content in MnO x Nanowires for Superior Supercapattery Performance
Source: ACS Omega. 2026 Feb 27;11(9):15337–53. doi: 10.1021/acsomega.5c12806 (PMC12980181; doi:10.1021/acsomega.5c12806)
Supplement: Supplementary file 1 [file ao5c12806_si_001.pdf]

Supplementary information

Tailoring Cobalt Content in MnO_x Nanowires for Superior Supercapattery Performance

Fernando José Soares Barros,[a*] Samuel da Silva Eduardo,[b] Klebson Lucas Pereira Cardozo,[a] Hector A. Vitorino,[c] Carlos Martins Aiube,[d] Mariana Lumi Ichihara Sado[e] Camila de Lima Ribeiro,[e] Paulo Eduardo Narcizo de Souza,[f] Alysson Martins Almeida Silva,[e] Auro Atsushi Tanaka[a*]

[a] Department of Chemistry, Federal University of Maranhão, Av. dos Portugueses, 1966, 65080-805, São Luís, MA, Brazil.

[b] Department of chemistry, Universidade Federal do Rio de Janeiro, Avenida Athos da Silveira Ramos, nº 149, 21941-909, Rio de Janeiro, RJ, 21941-901, Brazil.

[c] Department of Fundamental Chemistry, Institute of Chemistry, University of São Paulo, Av. Prof. Lineu Prestes, 748, São Paulo, SP 05508-000, Brazil.

[d] Institute of Chemistry, University of Brasília, Campus Universitário Darcy Ribeiro, Asa Norte, 70910-900, Brasília, DF, Brazil

[e] Department of Mechanical Engineering, University of Brasilia, Campus Universitário Darcy Ribeiro, Asa Norte, 70910-900, Brasília, DF, Brazil

[f] Institute of Physics, University of Brasilia, Campus Universitário Darcy Ribeiro, Asa Norte, 70910-900, Brasília, DF, Brazil

*Corresponding authors: fernando.barros@gpsa.ufc.br, tanaka.auro@ufma.br

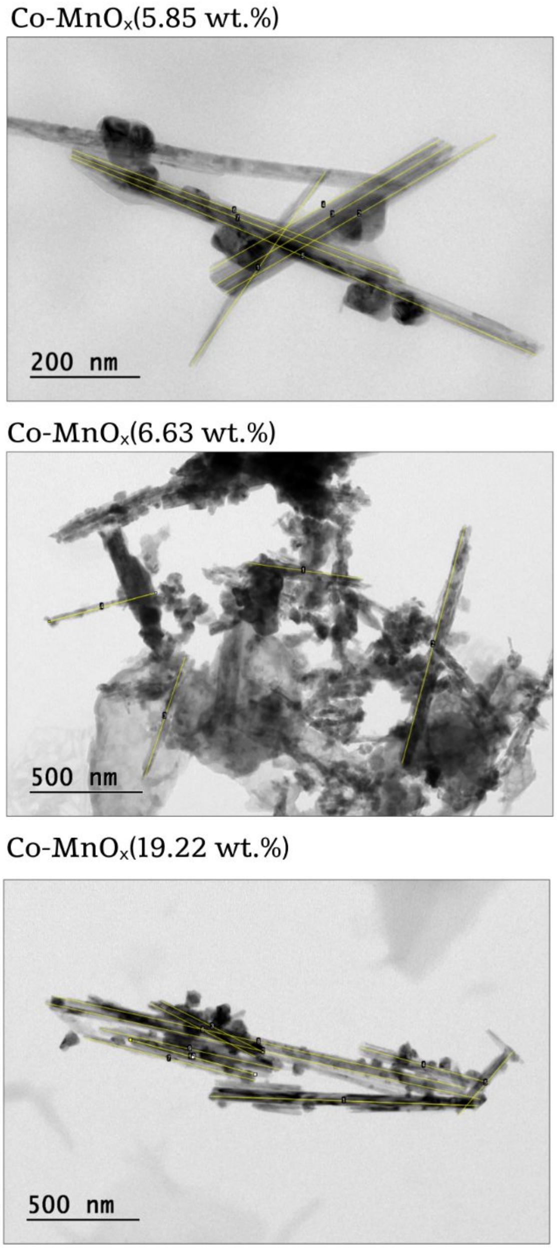

Figure S1: Representative TEM images of MnO_x nanowires analyzed using ImageJ for Co-MnO_x(5.85 wt.%), Co-MnO_x(6.63wt.%), and Co-MnO_x(19.22 wt.%).
